# Blast TBI Models, Neuropathology, and Implications for Seizure Risk

**DOI:** 10.3389/fneur.2014.00047

**Published:** 2014-04-09

**Authors:** S. Krisztian Kovacs, Fabio Leonessa, Geoffrey S. F. Ling

**Affiliations:** ^1^Laboratory of Neurotrauma, Department of Neurology, Uniformed Services University of the Health Sciences, Bethesda, MD, USA

**Keywords:** blast, traumatic brain injury, post-traumatic epilepsy, seizures, animal models, neuropathology, histopathology, tissue processing

## Abstract

Traumatic brain injury (TBI) due to explosive blast exposure is a leading combat casualty. It is also implicated as a key contributor to war related mental health diseases. A clinically important consequence of all types of TBI is a high risk for development of seizures and epilepsy. Seizures have been reported in patients who have suffered blast injuries in the Global War on Terror but the exact prevalence is unknown. The occurrence of seizures supports the contention that explosive blast leads to both cellular and structural brain pathology. Unfortunately, the exact mechanism by which explosions cause brain injury is unclear, which complicates development of meaningful therapies and mitigation strategies. To help improve understanding, detailed neuropathological analysis is needed. For this, histopathological techniques are extremely valuable and indispensable. In the following we will review the pathological results, including those from immunohistochemical and special staining approaches, from recent preclinical explosive blast studies.

## Introduction

Blast-related traumatic brain injury (TBI) is a frequent outcome of exposure to explosive device detonation. During the Global War on Terror (GWOT), which includes both Operation Iraqi Freedom (OIF) and Operation Enduring Freedom (OEF) in Afghanistan, the use of improvised explosive devices (IED), vehicle borne IED (VBIED), and improvised rocket assisted mortars (IRAM) resulted in a significant number of blast-related TBI (1–4). During the over 10 years of GWOT, almost 290,000 U.S. military personnel suffered TBI of which 68% was due to explosive blast exposure (5, 6).

The use of individual body armor systems (IBAS) reduces the incidence of lethal thoracic and abdominal combat related injuries dramatically when compared to previous wars when this protective equipment was not used. Thus, many soldiers survive who would not have had they not worn IBAS. An untoward consequence of increased survival is that blast-related TBI became more prevalent than in previous conflicts (2). These victims suffer a spectrum of neurological disorders ranging from subtle mild cognitive impairment, affecting the ability of a person to perform under demanding conditions, to severe disruption of brain function as serious as coma. These effects can be temporary or chronic. If the latter, they can have significant negative impact on patients and their families for decades at great emotional and economic costs to themselves and society.

The prevalence of epilepsy among GWOT TBI patients is unknown. From evidence derived from prior wars, it is expected that about 10–25% of patients with closed head TBI and over 50% of patients who have penetrating TBI will develop post-traumatic epilepsy (PTE) (7). The Department of Defense reports that 1.5% all combat related GWOT TBI are from penetrating injury (6). PTE can take any form of epilepsy but temporal lobe epilepsy (TLE) predominates with up to 62% of TBI patients suffering this type (8). It is important for clinicians to be aware that up to 15% of TBI patients from prior wars did not manifest seizures until five or more years after their injury (7). Recognizing this, the Veterans Administration (VA) has established a national network of Centers of Excellence for Epilepsy, which will provide long-term surveillance and care for these patients.

Most seizures occur within the first 2–3 years after the traumatic event, although the risk for developing PTE remains elevated for many years after injury. About 50% of patients, with even mild TBI (mTBI), who suffer early seizures, i.e., within the first 7 days after injury, will progress to PTE (9, 10). The highest risk for developing seizures correlates with TBI severity. Increased risk is associated with structural lesions such as dural penetration and intracranial hematoma. Findings from the Vietnam head injury survey show that cortical involvement, brain tissue loss, and intracranial retained metal fragments are high risk factors (11, 12).

During the last few years, the present and potential long-term impact of blast-related TBI among military personnel has fueled an increasing number of studies aimed to better understand the mechanisms of injury and characterize the pathobiology of blast-related TBI in order to improve its prevention detection and treatment. This effort is particularly relevant to combat related mTBI, where blast accounts for 72% of cases (13).

Confusion is often associated by the use of the words “primary” and “secondary” to define both physical causal mechanisms leading to injury and the neuropathology of the tissue response. In the case of tissue response, “primary” refers to the immediate tissue damage caused by the physical force such as tissue disruption from a blow to the head or a penetrating projectile as it traverses through brain parenchyma. Secondary injury relates to the pathophysiological response to the injury such as inflammation, excitatory amino acid release, or expression of reactive oxygen species. When referring to the physical explosive blast force causing TBI, primary refers to the direct injurious mechanism of the explosive blast wave. Secondary injury refers to TBI caused by being struck by material (bomb casing fragments, rocks, and dust) propelled by the blast, tertiary to the victim being physically thrown leading to an impact injury and quaternary for all other mechanisms, such as burns from fireball related burns, toxic fumes, radiation, etc. (14).

“Primary blast-induced injury” thus refers to the tissue damage caused by the explosive blast wave alone. A leading hypothesis for a primary mechanism for how explosive blast causes primary brain injury is that shock waves transit across the target tissue causing its acceleration and deformation. The extent of tissue damage depends on the shape of the blast shock wave, its peak overpressure and pulse duration, and the tissues’ natural resonant frequencies (3, 15, 16). The ideal blast shock wave can be represented by the Friedlander curve (17). The injurious effect of this primary blast mechanism is most significant in hollow organs including tympanic membranes, lungs, and gastrointestinal tract (18–26).

Another hypothesized primary mechanism is that shock waves impact the torso and are then transmitted to the brain causing TBI (27–31). In particular, it has been proposed that, indirect transmission of kinetic energy from the blast shock wave traveling through the large vessels of the body plays a key role in causing TBI. The blast overpressure compresses large body cavities, which creates oscillating waves inside the fluid contained in large abdominal and thoracic vascular vessels. The oscillating waves are conducted cephalad through these fluid columns into the brain resulting in both morphological and functional damage. Experimental data suggest that both direct (32) and indirect mechanisms (33) have important roles in the pathogenesis of blast TBI.

In order to test these proposed hypotheses and clarify the underlying pathophysiology in blast TBI, different preclinical methods have been developed using either shock or explosive blast tubes or open-field blast experiments (8, 19, 34). From these studies, extensive data has been amassed on blast shock wave–tissue interaction, blast exposure related cognitive, and behavior changes and brain pathology.

In this review, we focus on the pathology of blast-induced TBI from recent animal studies, summarizing gross and microscopic findings, tissue staining methods, and relevant neuropathology.

## Seizures and Epilepsy Following Traumatic Brain Injury

Epilepsy is a common disorder for which well-established and widely accepted animal models exist. These methods use either chemical or electrical approaches to induce seizures. For PTE study, injury is recreated using traditional experimental closed head TBI methods, such as fluid percussion injury (FPI) and controlled cortical impact (CCI), and penetrating head TBI, such as balloon inflation penetrating ballistic brain injury (PBBI) (35–39).

Acute TBI causes sudden changes in brain metabolism, blood flow, and homeostasis increasing the risk of immediate and chronic recurrent seizures (40, 41). One leading mechanistic hypothesis of PTE is contact between intracranial blood and the neuropil lowers seizure threshold (42). However, the conditions for closed head PTE are likely more complex and encompass a number of active TBI related processes. The physical forces causing head impact can create a variety of conditions favorable for seizures, such as acceleration, rotation, contusion and shearing of the blood vessels and fiber tracts, leading to hemorrhages, axonal injuries, gliosis, microglia activation, and Wallerian degeneration. Altered cerebral vasomotor regulation leading to blood flow disturbances, intracranial pressure changes, and altered vascular permeability can potentially contribute to by increasing extracellular calcium, glutamate, and reactive oxygen species formation. Iron from hemoglobin and transferrin accumulates in the brain as hemosiderin enhances the formation of toxic free radicals (40, 42, 43). Disrupted fiber tracts results in anterograde transynaptic neuronal degeneration with the loss of inhibitory interneurons thus lower seizure threshold. Release of aspartate, glutamate, and activation of NMDA receptors with reactive gliosis may also be contributing causative events leading to PTE (44). The size of the injury and the underlying pathophysiology can also alter the occurrence and intensity of non-convulsive seizures (NCS).

For penetrating TBI, PBBI generates more delayed and sporadic seizures compared to infarction following permanent middle cerebral artery occlusion (MCAO), a model of a stroke, with more acute and intense NCS soon after the injury (37, 45). There is a correlation between the volume of the infarct and the NCS activity in the MCAO model but the volume of the lesion after PBBI does not correlate with the seizures. However, there is a positive correlation with the ballistic kinetics of the PBBI and the size of the cavity created by different sizes of the inflated balloon (i.e., 5, 10, 12.5% PBBI) (46). It appears that seizure activity is sensitive to both the size of the injury and the ballistic kinetics and there is a significant difference in the timing and intensity of NCS after MCAO and PBBI. The results of these studies suggest that injury-specific treatment strategies need to be considered.

Histopathological findings in experimental models of PTE show similar changes to those of human TLE. Patients with TLE are usually classified in either the mesial TLE group or in the lateral or neocortex TLE group. Mesial structures of the temporal lobe with epileptogenic potential are the hippocampus and occasionally the amygdala and the entorhinal cortex (47, 48). Interestingly, histology analyses of hippocampal tissues from TBI patients with blunt head trauma or acceleration injury show similar cellular and structural changes compared to the pathology from non-trauma patients with TLE. Histology reports from patients operated on for TBI or drug-refractory TLE show direct hippocampal contusion, hippocampal sclerosis, and neuronal cell loss in the CA1–CA4 sectors with relatively mild histological changes in CA2 and the dentate gyrus. In a patient population with prolonged survival following head trauma the neural cell loss was significant in all hippocampal pyramidal cell subfields (49). The hippocampal degeneration appears to be progressive in nature revealing more severe neuropathological alterations in patients surviving more than 6 months than in patients with <1 week survival (50–53). Reactive astrogliosis is also detectable in TLE with increased expression of glial fibrillar acidic protein (GFAP). Blood–brain barrier (BBB) opening can lead to astrocyte activation through albumin-mediated transforming growth factor β (TGFβ)-dependent signaling (54–56). Other neuropathological findings in surgical specimens from patients with TLE described granule cell dispersion and temporal lobe sclerosis (47). Other, less frequently affected regions of the brain include selective neurons of the thalamus, basal forebrain, cerebellum, and brain stem (57, 58).

In animal models, FPI causes mossy fiber sprouting demonstrated by Timm staining in the ipsilateral hippocampus in rats with the loss of dentate hilar neurons (35). CCI generates common seizure risk factors in the brain, such as epidural hematoma, subdural hematoma (59), cell loss in the cortex and hippocampus, and neurogenesis in the dentate gyrus (60–63). CCI also results in mossy fiber sprouting in the dentate gyrus ipsilaterally in mice with concurrent late spontaneous post-traumatic seizures similar to human TLE (36). Indeed, the hippocampus seems to be one of the primary sites in epileptogenesis as there is increased acetylcholinesterase staining in human temporal lobe seizure specimens, especially in the outer portion of the molecular layer of the dentate gyrus (64).

Interestingly, whereas various experimental blast methods and studies exist to investigate brain injury, none have reported seizures. This raises the issue whether blast causes neuropathology that is distinct from blunt force. Blast waves can create similar neuropathological changes in the brain, most specifically in the hippocampus, as those observed in experimental PTE animal models. There is also evidence of neurodegeneration, axonal injury, and astrocytosis in the molecular layer of the hippocampus and the dentate gyrus at various short and long-term survival times (65–69). One confounding experimental issue is use of anesthetic agents when performing blast experiments. This is to provide humane treatment to subjects but may have the unintentional effect of suppressing spontaneous seizure activity. Furthermore, studies of reduced seizure threshold have not yet been reported. It should be noted that explosive blast study is a relatively new area of neuroscience research. The primary focus of these early blast studies has been to characterize the underlying physical mechanisms and pathophysiology that causes brain injury and not yet the development of PTE. Thus, these studies are limited largely to neuropathological and behavioral evaluations. Moreover, applying well-developed animal models for PTE, such as CCI or LFP, creates more reproducible injury and neuropathological changes in the brain. Injuries caused by CCI, LFP, or PBBI devices could be more circumscribed and focused to the brain area of interest that trigger PTE. Finding an ideal and reliable experimental blast TBI model with equivalent well-established characteristics of brain injury is still in progress. It is clear that more research is needed to study the relationship between blast TBI and PTE (50).

Animal models are useful in elucidating mechanisms underlying and structural alterations associated with PTE. They provide a rational basis by which more effective treatments may be developed. It is also important to be familiar with these experimental methods because the results of these studies, especially the observed histological changes in the central nervous system, provide a deeper understanding of the underlying pathophysiologies of the various types of TBI. It must be noted again that none of these traditional models use explosive blast. Thus, the insights gained from these animal models may be limited as they pertain to combat related explosive blast TBI.

## Experimental Models of Blast-Induced Neurotrauma

Appropriate clinical and military-relevant experimental animal methods are essential to characterize injuries and disorders of blast TBI. The injury model should be reproducible with a clearly identified injurious component simulating the features of human blast TBI. Injury severity should be predicted by the different mechanical properties of the injurious agent and the determined end-points of injury should be reflected by the chosen injurious component of the blast (28).

Various test methods are used to model explosive blast injuries suffered by humans. The most frequently used experimental models are open-field blasts, blast tubes, and shock tubes (28). An open-field blast is when an explosive device is detonated in an open area. It may be suspended above or placed directly on the ground. Subjects are located a specific standoff distance away from the device. This is the most accurate representation of the human condition. However, as in actual IED blasts, the shock waves produced are complex as they are subject to reflection off the ground and other surfaces. The fireball and debris cloud may contribute to the injury. Thus, it is difficult to study primary blast effects alone using this approach. For that reason, tubes are used.

In explosive blast tube experiments, a blast wave (shock wave plus blast wind) is created by the detonation of an explosive charge. The advantage of this approach, as compared to open-field blasts, is that equivalent blast intensities at the target can be achieved with significantly smaller explosive charges. Moreover, the experimental setup allows for the exposure of experimental subjects to a “pure” blast event without reflected shock fronts from the ground or other surfaces. Isolation of the primary blast mechanism is facilitated by adequate immobilization (to minimize tertiary mechanism), using uncased explosive (to prevent secondary mechanism), and placement of the subject beyond the detonation fireball (to avoid quaternary mechanism). Examples of blast tubes are the tube developed by Parks used by Bauman et al. (67) and De Lanerolle et al. (70) to study blast-induced TBI in swine, and the Clemedson tube (71) used in Sweden to study the blast-induced TBI in rat (72, 73).

The tube developed by Parks is 70-feet long, open at both ends and has three sections: a 6-feet long heavy walled driver chamber (where the explosive is detonated) with a diameter of 34″, a 10-feet expansion cone, and a 50-feet test section, with a 6-feet diameter. The standoff distance is typically 15–25 feet.

The Clemedson tube is much smaller (about 1.5 m in inner length), closed at the detonation end, represented by a conical shaped chamber about 0.57 m deep. The test section (<1 m long) is cylindrical, with an inner diameter of 0.4 m. The standoff is about 1 m. Two consequence of the difference in size and standoff is that the Clemedson tube can be used only for smaller animals and that blast pulse durations will be shorter.

Obviously, a method using an explosive is the most accurate way to study explosive blast effects. However, there are significant practical considerations when using these blast tubes. Requirements include specialized testing locations (usually, ranges), personnel specifically trained in the safe use of explosives, and expense associated with these. In addition, explosive blasts, whether in the open-field or in a tube are typically carried out in an outdoor setting and are consequently subject to weather and other environmental conditions (74).

Shock tubes using compressed gas, such as helium, as opposed to explosives are an alternative to blast tubes. They are safer, more cost effective, and can be used indoors. These tubes are smaller than explosive-driven tubes and are closed at one end. They consist of a “driver” section at the closed end, separated from a “driven” section by a frangible or breakable diaphragm composed of mylar or cellulose acetate. The process begins with the generation of high pressure by the pumping of gas within the closed off driven section. When the pressure reaches a critical level, the diaphragm ruptures creating a shock wave. The shock wave characteristics can be controlled or tuned by changing subject standoff from the diaphragm, varying the membrane material or thickness, changing the shape of the closed end of the driver, and using different gases to pressurize the membrane. Similar to blast tubes, most shock tubes are designed to contain the subject animal within their “driven” section. Examples are those used at Walter Reed Army Institute of Research (33), the University of Kentucky in Lexington (75), Wayne State University (76), and Johns Hopkins University (77). In addition, there are smaller models of shock tube that are designed to generate a shock wave to impact a target outside the tube itself so as to study the effect on a specific body region, such as the head or the chest (30, 33, 65, 66, 78–83). Examples of this latter type are those used at the Florida Institute of Technology and Banyan Biomarkers, Inc. (66) and the University of Toronto (83).

Shock tubes have their own important drawbacks. Very importantly, the physics of the gas-driven shock waves may differ from explosive shock waves. If so, the injury pattern produced may not be comparable to the human condition. Gas-driven shock waves are often atypical, showing an apparent pressure plateau following the initial pressure peak. This is likely due by the existence of two successive pressure waves; the first directly coming from the bursting diaphragm and the second reflected back from the tube end. A single and more typical-looking pressure wave is obtained by allowing sufficient standoff, which permits the reflected wave to reach and fuse with the direct one. Another issue is the possible impact of diaphragm fragments on the subject. Even low mass fragments, when accelerating at high rates, will exert significant force on subjects, which means the resultant injury is not primary blast effect alone. Finally, the physical load of multiple small fragments may affect the dynamics of body–head acceleration.

A common issue of both explosive-driven and gas-driven shock tubes is the jet stream effects created near the tube exit. This jet stream creates an unrealistic dynamic pressure effect that can be avoided by placing the target sufficiently far from the tube’s exit or, in the case of external exposure, sufficiently off axis to the tube’s nozzle (66, 83).

Both explosive and gas-driven shock tubes aim to recreate primary blast conditions with ideal Friedlander waves. Real world exposures are more complicated as reflected shock waves create a complex interaction with primary shock waves. To replicate war related conditions, some investigators have carried out studies using surrogates of military vehicles, buildings, or bunkers (67, 68, 74, 84, 85). Each is appropriate for recreating real world condition but methodological differences interfere with generalization of results (74).

Finally, rodents, pigs, rabbits, and non-human primates (NHPs) used for blast studies widely differ in their neuroanatomy and neurophysiology, which can further contribute to the variations in the observed pathological and physiological changes of experimental blast injuries (28, 74).

## Pathology of Blast-Related Brain Injury

Recent studies have identified candidate pathophysiological processes that likely play key roles in the genesis of blast TBI. From detailed histopathological analyses, common findings include small and larger intracranial hemorrhages, edema, vasospasm, neuronal damage/degeneration, focal or diffuse axonal injury, glial cell activation, and inflammatory reactions (1, 86). Optimizing identification of tissue injuries is highly dependent on using the most appropriate histological methods and stains as well as on timing after injury ictus and sampled brain region. For general morphological examinations (neuronal injury, cell death, intracranial hemorrhages, edema formation, and inflammation) hematoxylin and eosin (H&E) and cresyl-violet are used. Luxol-fast blue, a special myelin stain, is used routinely for myelin damage. For the detection of more subtle cellular changes, immunohistochemistry (IHC) is the general method. One of the most widely examined features of TBI is diffuse axonal injury. Traditionally, axonal injury is detected by silver staining or β-amyloid precursor protein (β-APP) IHC (87–90). GFAP and various microglia stains are used to label activated astrocytes and microglia cells (91–93). For ultrastructural examinations at the subcellular level electron microscopy is the preferred method.

As part of the research program PREVENT (Preventing Violent Explosive Neurotrauma), Baumann et al. use a swine model and the Parks explosive-driven shock tube to study explosive blast TBI (67). Within the tube the pigs are restrained in a sling that minimizes movement during the blast, and exposes subjects side-on to the blast. In addition, these investigators use both a surrogate military vehicle and 2-room building so as to recreate more typical complex shock waves. Brain specimens are obtained at 2 weeks after blast exposure. For axonal injury, a modified Gallyas silver method, as made available by FD Neurotechnologies, is used (94). This staining technique labels injured/degenerating axons and neurons as early as 24 h after injury. IHC is used to label cells positive for GFAP as well as other markers. Silver staining reveals degenerated axons in the ipsilateral white matter tracts of corona radiata and cerebellum. Astrocyte activation is evident in the ipsilateral white matter of the cortex and in multiple layers of the ipsilateral hippocampus. Elevated GFAP, neuron specific enolase (NSE), and myelin basic protein (MBP) expression are also detected 6, 24, and 72 h after exposure. Additional observations include changes in the electroencephalogram (EEG) patterns, vasospasm in carotid artery branches, and disturbances in the movement of the pigs involving major joints and limbs (knees and metacarpals). Detailed neurological function assessment is made using motion analysis technologies for gait, EEG telemetry, spatial memory testing, and cerebral angiography. However, anatomical differences between swine and human skulls can generate discrepancies in the interpretation of biological and biomechanical events.

A similar approach to blast exposure is used in a swine study carried out by de Lanerolle et al. (70). Specimens are collected 72 h and 2 weeks after blast. Paraffin-embedded sections are used for standard and immunohistochemical stainings: H&E, Luxol-fast blue, Fluoro-Jade B (neurodegeneration), GFAP, β-APP, and CD68 (macrophage/microglia marker).

Analysis reveals very limited neuronal injury with Fluoro-Jade B failing to reveal positive cells. Intracranial hemorrhages and fiber tract demyelination are not present. Dark, shrunken neurons are noticed but since they are also seen in controls, their presence is attributed to mechanical manipulation of the tissue. Red (eosinophil) neuronal degeneration is occasionally visible throughout the neural tissues both in blast and sham control animals. β-APP IHC is positive in the periventricular white matter close to the lateral ventricle in all groups. The axonal injury, around or close to the ventricles, is explained by a fluid-tissue interface effect generated by local pressure transients at the site, or ventricular volume increase strong enough to cause axonal deformation. GFAP activity is also enhanced in the different layers of the hippocampus and cortical gray and white matters. Their morphology is different from those activated by neuronal injury and the number of activated astrocytes in the hippocampus is significantly higher in the animals exposed in the vehicle or the building. Microglia activation is visible in the central white matter and corpus callosum. One explanation of the glial activation is the transient opening of the BBB triggering the activation of astrocytes by extravasated albumin resulting in excitatory neuronal injury. These findings together support the notion that astrocytosis and periventricular axonal injury may have an important role in the potential for long-term TBI exacerbations, mood, and cognitive disorders.

Lu et al. report their NHP study using open-field blast with either single or double-blast exposure (68). The outcome of exposure to the following conditions is evaluated and compared: single-blast at 80 kPa (equivalent to 11 psi; SBL), single-blast at high intensity at 200 kPa (equivalent to 29 psi; SBH), and double-blast (DBL) at 80 kPa. In the DBL group, exposures are carried out 3 days apart. Specimens are obtained at either 3 days or 1 month post-blast. General morphological analysis uses H&E and TUNEL for apoptosis. IHC is used for the detection of S100B and GFAP (for astrocyte reaction), MBP, neuronal nuclear antigen (NeuN), β-APP, aquaporin-4 (AQP4, for water channel identification), and oligosaccharide-specific agglutinin I anti-lecithin antibody (to reveal microglia cells). Electron microscopy is also performed in order to detect ultrastructural changes.

At gross pathological examination, no visible damage can be detected in the brain, and only minor injuries are noticeable in the lungs. MRI only detects a right anterior lobe cerebellar lesion in a single subject. Microscopically, there are neuronal cell changes in the cortex, the cerebellar Purkinje-cells and the hippocampus such as dark, shrunken neurons with distorted dendrites in all groups with elevated NeuN reaction. Apoptotic cells are rarely co-labeled with GFAP and MBP in the subcortical areas 1 month after injury. The number of apoptotic cells is increased and MBP reaction is reduced in the SBH and DBL groups. Increased β-APP reaction is also observed in the neuronal perykarion and around axons. Besides the neuronal alterations in the cerebellum, the astrocytes show reactive changes in the SBH and DBL groups by S100, GFAP, and AQP4 staining. Electron microscopy on tissues from the cerebellum reveal structural damages in the nucleus, mitochondria, and cytoplasmic filaments of the Purkinje-cells, with the formation of stacks of smooth endoplasmic reticulum, myelin sheath degeneration, astrocyte filamentous and end-feet hypertrophy, microglial activation, and severe oligodendrocyte cell injury. Interestingly, vascular changes are observed in the cerebellum, with obliterated and collapsed capillaries, endothelial cytoplasm vacuolations and accumulation of perithelial cells.

These pathological results correlate with observed behavioral changes in motor coordination and working memory. The lesion detected by MRI shows widespread pathology in the above described area suggesting the vulnerability of the cerebellum. The accumulation of the smooth endoplasmic reticulum in the Purkinje-cells can be a part of a protective mechanism by calcium sequestration. Furthermore, damage to the oligodendrocytes, astrocytes, and capillaries likely contribute to cognitive, motor and other neurological dysfunctions, brain edema, and ischemic-hypoxic damage. Although the study provides a broad pathological overview in blast TBI, the sample size is relatively small and further long-term behavioral studies are required to define neurological deficits.

To determine whether or not torso IBAS mitigates of TBI, Long et al. use a compressed air-driven shock tube to create blast injury in chest-protected and unprotected rats (33). Chest protection is a Kevlar vest that completely covers the rat’s thorax but leaves the head exposed. The animals are placed in a transverse prone position in a wire-mesh holder across the mouth of the shock tube. Brain samples are collected 2 weeks after blast exposure. Brains are cresyl-violet, thionine, and silver-stained. The observed pathological alterations are torso protection and intensity dependent. Neural cell loss is observed, along with gliosis, fiber degeneration, hemorrhage, and necrosis, in the brain of unprotected rats exposed to 147 kPa (equivalent to 21 psi), but not 126 kPa (equivalent to 18 psi) blasts. These changes are more severe in the hemisphere facing the blast. Brains from rats exposed to the lower blast show extensive silver-stained fiber degeneration that is bilateral.

Chest protection does not affect the pathological outcome in 147 kPa blast – exposed rats but largely prevents fiber degeneration in the brains of animals exposed to 126 kPa blasts. No evident pathology is observed in the brains at the lowest blast intensity level (114 kPa or 16 psi). These findings suggest that chest protection does contribute to TBI mitigation, particularly at lower blast intensities. Furthermore, these observations lend further support to that of prior studies (31) that the second hypothesized mechanism of how blast injures brain may be valid.

Studying head and torso protection, Koliatsos et al. use a shock tube generating overpressure with compressed helium, with mice placed inside the shock wave tube fixed in a wire-mesh holder disallowing body or head motion (95). The torso and/or head of each mouse are protected by a Plexiglas cover. Animals are exposed to different blast intensities either in a prone or supine position. Social recognition, spatial memory, and motor coordination outcome measures are used. Brains are collected at 1, 3, 5, 7, and 14 days after exposure. Internal organs – lungs, liver, heart, spleen, and kidney – and eyes were also examined. Standard formalin immersion-fixed paraffin-embedded, and perfusion fixed frozen tissues are stained with routine H&E, various special and immunostains (cresyl-violet, Mallory trichrome, elastic fiber stain, Fluoro-Jade, APP, phosphorylated neurofilament, and TUNEL). FD Neurosilver kit is used to detect axonal injury.

Findings are injuries to the internal organs (lung, heart, liver, kidney, and spleen) that are mainly hemorrhages and hemorrhagic infarcts. These correlate with blast wave intensities and body position. Neuropathological results of blast at lower blast intensity include extensive silver-stained axonal injury involving the cerebellum, brainstem, corticospinal tracts, optical, and auditory pathways. Axonal injury is more prominent 14 days after the exposure. No histological reactions are detected in animals at 1, 3, and 5 days after blast. Special stains fail to reveal any brain hemorrhage, neuronal injury, or cell death. Occasional APP and phosphorylated neurofilament positivity is visible in the corpus callosum and anterior vermis. Interestingly, when torso protection is applied, there is no observable white matter tract degeneration and no behavioral deficits.

The finding that torso protection is neuroprotective in blast, especially against diffuse axonal injury, has both important clinical and mechanistic implications. These findings, consistent with those reported by Long et al. (33), point to the likely role of the second mechanism of blast TBI, which is blast chest compression and vascular cephalad conduction of shock waves into the brain. Clinically, this supports the military’s use of IBAS as likely helping to protect service members from both blast-related torso and brain injuries.

The importance and usefulness of silver staining is further emphasized and convincing in work by Garman et al. (65). As part of the PREVENT blast program, they conduct an initial neuropathological characterization in body protected rats exposed to blast. Animals are positioned in a helium-driven shock tube within a wedge-shaped holder protecting the torso but leaving the head exposed. Besides protecting the torso, the holder increases the intensity of the shock wave at the target, by creating a mach stem along the side of the wedge. To prevent gross motion, the head is held in place with a leather sling. The shock tube generates a peak pressure of 35 psi, resulting in 25% mortality from apnea. Brains are collected at 1 and 3 days and then 2 weeks. H&E, de Olmos amino cupric silver, and immunostains for GFAP, ionized calcium-binding adapter molecule 1 (Iba1) and CD68 (for microglia activation), APP, and IgG (for brain edema) are performed. Not surprisingly, silver staining is the most sensitive method in identifying TBI, labeling axonal damage as well as neuronal degeneration.

Neuronal degenerations including axons and dendrites are the most prominent histological alterations during the first 2 weeks in blast-exposed rats with body protection. Degenerating neuronal cell bodies are most detectable at 1 and 3 days showing a scattered distribution with some preference in various cortical regions, CA1 pyramidal layer of the hippocampus and the cerebellar cortex, the latter suggesting synaptic or terminal degeneration. The axonal damage marked by silver staining is prominent at all-time points, but most evident after 2 weeks, affecting both sides of the brain except for the entorhinal cortex and hippocampal dentate gyrus, which show stronger contralateral reaction. This is believed to be caused by a diffraction effect or localized shock amplification on the contralateral side of the skull or by the effect of diffraction coupled with skull flexure. The injured fiber tracts include various long tracts such as the optic tract, internal and external capsules, thalamic pathways, cerebral and cerebellar peduncules, trigeminal tracts, and pyramids. APP-based detection of axonal injury is minimal. There is no astroglial reaction and only weak microglia activation is visible adjacent to brain regions with neuronal degeneration. Breach of the BBB using IgG is only seen in the 1 day group mostly on the contralateral side of different brain regions.

This study demonstrate that, in this blast model, silver staining was more effective in revealing axonal injury than APP, a marker which is most prominently detected in axonal injuries related to acceleration/deceleration mechanisms (96, 97). This study also provides evidence of blast-related breach of the BBB. However, its relation with axonal injury, if any, is unclear.

A study by Goldstein et al. (69) examines the connection between blast-induced TBI and chronic traumatic encephalopathy (CTE). Neuropathological examinations of four military veterans who died in blast or concussive injuries show similar brain changes as four athletes who suffered concussive injuries in football, wrestling, etc. The image is correspondent with CTE, a tau protein-related neurodegenerative disease (98–101). These human neuropathological observations are compared with the pathological outcome of mice exposed to blast. In this model, mice are placed prone within a shock tube. Only the heads are exposed, side-on, to the gas-driven shock wave as the rest of the body is protected within the holding fixture. Heads are not secured for some subjects, which allow testing of the hypothesis that blast-induced head acceleration contributes to TBI. The blast is reported to be comparable to detonation of 5.8 kg trinitrotoluene (TNT). Measurements of intracranial pressure at the time of shock wave impact confirm the intracranial transmission of stress waves occurs without significant contribution of torso-transmitted shock waves. Brains are collected at 2 weeks post-blast, saline perfused, prefixed in 10% neutral-buffered formalin, block-sectioned, and post-fixed in 4% paraformaldehyde. Serial sections are cut from paraffin-embedded blocks and stained with various stains including IHC for axonal injury, tau pathology, astrocytosis, and cholinergic motor neurons. Brain tissues are also processed for ultrastructural examinations.

Gross examinations of the brains do not show any visible macroscopic tissue injury. By histological examinations single-blast exposure produces CTE-like changes in the mouse brain such as tau protein immunoreactivity, phosphorylated tau proteinopathy, cortical and hippocampal neurodegeneration, permanent perivascular pathology, myelinated axonopathy, and chronic neuroinflammation with astrocytosis and microgliosis. Blast produces “dark neurons” in close proximity to abnormal capillaries (102, 103). Moreover, axonal conduction velocity is reduced in the hippocampus and synaptic transmission disturbances resulting in learning and memory deficits. Head immobilization prevents blast-induced hippocampus-related behavioral deficits. Electron microscopy verifies persistent microvascular pathology and astrocyte end-feet swelling suggesting BBB compromise, which in turn possibly plays a role in local hypoxic, inflammatory, and neurodegenerative changes.

The similarities between human CTE cases and the experimental method described above suggest that different scenarios can induce a common pathway leading to similar morphological changes. The results from this mouse blast study are consistent with the morphological, neurophysiological, and cognitive deficits that are reported in military veterans and athletes with blast and/or concussive-related CTE. In addition, this study is also significant because it suggests that head acceleration plays a critical role in TBI.

## Summary and Discussion

In this review, we provide a brief review on experimental models of brain trauma, development of PTE and the pathological/histological features of TBI, including blast. Our intent is to give the reader an overview of the most routinely used and reproducible histopathological methods and neuropathological results published on blast TBI and PTE as these represent the cellular basis of this injury and its clinical consequence, such as seizures. It is at this level that rational comparisons may be made among the different TBI types as well as, very importantly, between preclinical models and the human condition. Increasing demand in the field of blast TBI to understand the physics and pathophysiology of blast-related brain injury has produced a large number of scientific publications reporting, sometimes contradictory, results obtained from animal studies (28, 86). These reports provide information about both morphological alterations in the CNS and also neurophysiological and behavioral aspects of blast injury. While it is extremely important to examine blast TBI and its consequences in every respect, pathological evaluation is probably the ultimate way to prove or disapprove mechanistic theories.

The pathological methods and results reviewed above underpin several technical issues, which need to be taken into consideration when working with tissue specimens, especially brain. The most important is to be able to recognize tissue and cellular changes and responses to a noxious event. It is one of the most crucial rules to learn to recognize common artifacts in CNS tissues, which are of no pathological significance (104). Failure to do so will lead to conclusions that are misleading and erroneous (105–108). Artifacts can be caused by improper tissue handling that, many times, are unavoidable (109) but following current guidelines could help to overcome these potential technical issues. Nevertheless, some of them are worth mentioning (110, 111). Microscopically, underperfused brain tissues demonstrate collapsed microvessels containing blood, with tissue retraction around them and dark, basophilic neurons are readily observable. These artifacts make histological interpretations difficult. Not all parts of the brain will necessarily be evenly well-perfused, but a good perfusion should produce distended vessels throughout the brain with no or minimal artifacts (110, 111).

One of the most frequently noted and long-debated artifacts in surgical human specimens and various experimental studies is the “dark neuron,” which is often interpreted as neuronal degeneration or death (112, 113). Neurons are highly susceptible to ischemic/hypoxic injuries that can be detected microscopically after 6–12 h in humans and 30–90 min in experimental animals (104, 114). The cytological hallmark of neuronal injury is the eosinophilic degeneration or “pink neurons.” These cells are shrunken with eosinophilic cytoplasm, glassy, basophilic pyknotic nucleus, and absent Nissl substance. After dead neurons and cell debris have been phagocytosed, glial cells appear and proliferate creating a glial scar tissue. Axonal transection, most frequently in lower motor neurons, can produce central chromatolysis when the cell itself is intact, the cell body is rounded and the nucleus and Nissl substance is displaced peripherally (104, 114, 115). Apoptotic, fragmented cells are easily recognizable even for the inexperienced eyes. “Dark neurons” on the other hand, have a shrunken angular cell body with deeply stained cytoplasm, small, irregular, dark basophilic nucleus with loss of details. Dendrites often have a characteristic cork-screw shaped appearance. Such neurons are more frequent in immersion-fixed brains but adequately perfused material can still contain numerous dark neurons in experimental neuropathology (110, 111, 116, 117). Mechanical post-mortem manipulation of the brain can increase the number of these neurons (110, 118). Interestingly, the presence of these contracted neurons has been reported in some acute neuropathological states making the distinction between true neuronal degeneration and artificial dark neurons challenging (119–122). Although neuronal degeneration and cell death can be often detected on routine H&E stained slides, using special stains specific for neurodegeneration can significantly assist to recognize neuronal damage. Fluoro-Jade B and Fluoro-Jade C are both recommended in the identification of neuronal degeneration and the degeneration of fine neuronal processes (111, 123). Silver staining has an important role in experimental neuropathology to detect axonal injury. Even if β-APP fails to label injured axons, silver techniques can help to detect early, and more often, late axonal degeneration (65, 95). Mastering any of the silver staining technique can be challenging but commercially available silver stain kits are easy to use and reliable. In general, for most neuropathological experimental studies, a set of special and immunohistochemical basic stains can provide an initial step toward a close evaluation of the tissue samples. The usage of negative and, ideally, positive tissue controls is of the utmost importance. Finally, it can’t be overemphasized that experiments, TBI or others, involving morphological evaluations should be reviewed by experienced morphologists or pathologists to avoid further inconsistencies among researchers (111, 124).

Fortunately, most researchers working with neural tissues are using appropriate current pathological methods but future investigators in the field, especially those without a background in pathology, should take into consideration the above discussed technical details. Moreover, the validity of some of the methods used to reproduce blast phenomenon may be lacking. The heterogeneity of results may be partly the result of this inadequacy and partly reflect differences in experimental designs. When considered in balance, the collective work still reveals important insights on mechanism of blast-related injury.

Some key findings are that explosive blast, when of sufficient severity, leads to brain pathology. The most consistent neuropathological findings are multifocal axonal and neuronal injuries detected by silver staining, astroglial alterations, inflammation with elevated cytokine and reactive oxygen species activity, BBB anomalies, and intracranial hemorrhages. This pathology correlates with behavior changes such as spatial and cognitive performance and coordination. Very important clinically is the evidence supporting the benefits of body armor in mitigating blast TBI as well as torso protection. This also provides supporting evidence to the notion that caudal transmission of shock waves through the thoracic and intracranial blood vessels plays a role in TBI genesis. Also very important is the demonstration that torso protection also mitigates diffuse axonal injury. The role of primary blast in causing TBI is still unclear. However, it does appear that head acceleration is an important contributor to TBI as well.

Seizures are an important clinical consequence of all TBI. Although the precise impact of this clinical condition on explosive blast TBI recovery is still being elucidated, the finding that explosive blast leads to consistent neuropathological brain changes raises significant concern that seizures and epilepsy may be more prevalent than previously suspected. Fortunately, the VA is taking a comprehensive prospective longitudinal approach to study PTE in blast TBI victims.

## Conflict of Interest Statement

The authors declare that the research was conducted in the absence of any commercial or financial relationships that could be construed as a potential conflict of interest. The opinions and views expressed herein belong only to those of the authors. They are not of and should not be interpreted as being endorsed by the Uniformed Services University of the Health Sciences, Department of Defense or any other agency of the U.S. government.

## References

[1] Ling G Bandak F Armonda R Grant G Ecklund J Explosive blast neurotrauma. J Neurotrauma (2009) 26(6):815–25 10.1089/neu.2007.0484 19397423

[2] Ling GS Ecklund JM Traumatic brain injury in modern war. Curr Opin Anaesthesiol (2011) 24(2):124–30 10.1097/ACO.0b013e32834458da 21301332

[3] Magnuson J Leonessa F Ling GS Neuropathology of explosive blast traumatic brain injury. Curr Neurol Neurosci Rep (2012) 12(5):570–9 10.1007/s11910-012-0303-6 22836523

[4] Okie S Traumatic brain injury in the war zone. N Engl J Med (2005) 352(20):2043–7 10.1056/NEJMp058102 15901856

[5] Masel BE Bell RS Brossart S Grill RJ Hayes RL Levin HS Galveston Brain Injury Conference 2010: clinical and experimental aspects of blast injury. J Neurotrauma (2012) 29(12):2143–71 10.1089/neu.2011.2258 22655746

[6] AFHS Center. DoD TBI Statistics 2000-2013. Washington, DC: Department of Defense (2013) p. 1–5

[7] Chen JW Ruff RL Eavey R Wasterlain CG Posttraumatic epilepsy and treatment. J Rehabil Res Dev (2009) 46(6):685–96 10.1682/JRRD.2008.09.0130 20104398

[8] Morganti-Kossmann MC Yan E Bye N Animal models of traumatic brain injury: is there an optimal model to reproduce human brain injury in the laboratory? Injury (2010) 41(Suppl 1):S10–3 10.1016/j.injury.2010.03.032 20416875

[9] Temkin NR Risk factors for posttraumatic seizures in adults. Epilepsia (2003) 44(Suppl 10):18–20 10.1046/j.1528-1157.44.s10.6.x 14511390

[10] Temkin NR Preventing and treating posttraumatic seizures: the human experience. Epilepsia (2009) 50(Suppl 2):10–3 10.1111/j.1528-1167.2008.02005.x 19187289

[11] Raymont V Salazar AM Lipsky R Goldman D Tasick G Grafman J Correlates of posttraumatic epilepsy 35 years following combat brain injury. Neurology (2010) 75(3):224–9 10.1212/WNL.0b013e3181e8e6d0 20644150 PMC2906177

[12] Salazar AM Jabbari B Vance SC Grafman J Amin D Dillon JD Epilepsy after penetrating head injury. I. Clinical correlates: a report of the Vietnam Head Injury Study. Neurology (1985) 35(10):1406–14 10.1212/WNL.35.10.1406 3929158

[13] Wilk JE Thomas JL McGurk DM Riviere LA Castro CA Hoge CW Mild traumatic brain injury (concussion) during combat: lack of association of blast mechanism with persistent postconcussive symptoms. J Head Trauma Rehabil (2010) 25(1):9–14 10.1097/HTR.0b013e3181bd090f 20051900

[14] Phillips YY Primary blast injuries. Ann Emerg Med (1986) 15(12):1446–50 10.1016/S0196-0644(86)80940-4 3535591

[15] Desmoulin GT Dionne JP Blast-induced neurotrauma: surrogate use, loading mechanisms, and cellular responses. J Trauma (2009) 67(5):1113–22 10.1097/TA.0b013e3181bb8e84 19901677

[16] Cullis IG Blast waves and how they interact with structures. J R Army Med Corps (2001) 147(1):16–26 10.1136/jramc-147-01-02 11307674

[17] Baker WE Explosions in Air. Austin: University of Texas Press (1973).

[18] Ritenour AE Blackbourne LH Kelly JF McLaughlin DF Pearse LA Holcomb JB Incidence of primary blast injury in US military overseas contingency operations: a retrospective study. Ann Surg (2010) 251(6):1140–4 10.1097/SLA.0b013e3181e01270 20485126

[19] Kocsis JD Tessler A Pathology of blast-related brain injury. J Rehabil Res Dev (2009) 46(6):667–72 10.1682/JRRD.2008.08.0100 20104396

[20] Wolf SJ Bebarta VS Bonnett CJ Pons PT Cantrill SV Blast injuries. Lancet (2009) 374(9687):405–15 10.1016/S0140-6736(09)60257-9 19631372

[21] Wightman JM Gladish SL Explosions and blast injuries. Ann Emerg Med (2001) 37(6):664–78 10.1067/mem.2001.114906 11385339

[22] Patterson JH Jr Hamernik RP Blast overpressure induced structural and functional changes in the auditory system. Toxicology (1997) 121(1):29–40 10.1016/S0300-483X(97)03653-6 9217313

[23] Mayorga MA The pathology of primary blast overpressure injury. Toxicology (1997) 121:17–28 10.1016/S0300-483X(97)03652-4 9217312

[24] Elsayed NM Toxicology of blast overpressure. Toxicology (1997) 121(1):1–15 10.1016/S0300-483X(97)03651-2 9217311

[25] Pennardt A Blast Injuries [Internet; cited 2014 Mar 28]. Available from: http://emedicine.medscape.com/article/822587-overview

[26] Langworthy MJ Sabra J Gould M Terrorism and blast phenomena: lessons learned from the attack on the USS Cole (DDG67). Clin Orthop Relat Res (2004) 422:82–7 10.1097/01.blo.0000128293.43913.ca 15187838

[27] Cernak I Savic J Ignjatovic D Jevtic M Blast injury from explosive munitions. J Trauma (1999) 47(1):96–103 10.1097/00005373-199907000-00021 10421194

[28] Cernak I Noble-Haeusslein LJ Traumatic brain injury: an overview of pathobiology with emphasis on military populations. J Cereb Blood Flow Metab (2010) 30(2):255–66 10.1038/jcbfm.2009.203 19809467 PMC2855235

[29] Courtney AC Courtney MW A thoracic mechanism of mild traumatic brain injury due to blast pressure waves. Med Hypotheses (2009) 72(1):76–83 10.1016/j.mehy.2008.08.015 18829180

[30] Cernak I Wang Z Jiang J Bian X Savic J Cognitive deficits following blast injury-induced neurotrauma: possible involvement of nitric oxide. Brain Injury (2001) 15(7):593–612 10.1080/02699050119009 11429089

[31] Cernak I Wang Z Jiang J Bian X Savic J Ultrastructural and functional characteristics of blast injury-induced neurotrauma. J Trauma (2001) 50(4):695–706 10.1097/00005373-200104000-00017 11303167

[32] Säljö A Arrhén F Bolouri H Mayorga M Hamberger A Neuropathology and pressure in the pig brain resulting from low-impulse noise exposure. J Neurotrauma (2008) 25(12):1397–406 10.1089/neu.2008.0602 19146459

[33] Long JB Bentley TL Wessner KA Cerone C Sweeney S Bauman RA Blast overpressure in rats: recreating a battlefield injury in the laboratory. J Neurotrauma (2009) 26(6):827–40 10.1089/neu.2008.0748 19397422

[34] Wang Z Sun L Yang Z Leng H Jiang J Yu H Development of serial bio-shock tubes and their application. Chin Med J (Engl) (1998) 111(2):109–13 10374367

[35] Kharatishvili I Nissinen JP McIntosh TK Pitkänen A A model of posttraumatic epilepsy induced by lateral fluid-percussion brain injury in rats. Neuroscience (2006) 140(2):685–97 10.1016/j.neuroscience.2006.03.012 16650603

[36] Hunt RF Scheff SW Smith BN Posttraumatic epilepsy after controlled cortical impact injury in mice. Exp Neurol (2009) 215(2):243–52 10.1016/j.expneurol.2008.10.005 19013458 PMC4694635

[37] Lu XC Mountney A Chen Z Wei G Cao Y Leung LY Similarities and differences of acute nonconvulsive seizures and other epileptic activities following penetrating and ischemic brain injuries in rats. J Neurotrauma (2013) 30(7):580–90 10.1089/neu.2012.2641 23234254

[38] Golarai G Greenwood AC Feeney DM Connor JA Physiological and structural evidence for hippocampal involvement in persistent seizure susceptibility after traumatic brain injury. J Neurosci (2001) 21(21):8523–37 11606641 10.1523/JNEUROSCI.21-21-08523.2001 PMC6762822

[39] Williams AJ Ling GS Tortella FC Severity level and injury track determine outcome following a penetrating ballistic-like brain injury in the rat. Neurosci Lett (2006) 408(3):183–8 10.1016/j.neulet.2006.08.086 17030434

[40] Evans RW Neurology and Trauma. 2nd ed New York: Oxford University Press, Inc (2006).

[41] Kharatishvili I Pitkanen A Posttraumatic epilepsy. Curr Opin Neurol (2010) 23(2):183–8 10.1097/WCO.0b013e32833749e4 20125011

[42] Willmore LJ Sypert GW Munson JB Recurrent seizures induced by cortical iron injection: a model of posttraumatic epilepsy. Ann Neurol (1978) 4(4):329–36 10.1002/ana.410040408 103489

[43] Payan H Toga M Berard-Badier M The pathology of post-traumatic epilepsies. Epilepsia (1970) 11(1):81–94 10.1111/j.1528-1157.1970.tb03869.x 4987163

[44] Saji M Reis DJ Delayed transneuronal death of substantia nigra neurons prevented by gamma-aminobutyric acid agonist. Science (1987) 235(4784):66–9 10.1126/science.3798095 3798095

[45] Mountney A Shear DA Potter B Marcsisin SR Sousa J Melendez V Ethosuximide and phenytoin dose-dependently attenuate acute nonconvulsive seizures after traumatic brain injury in rats. J Neurotrauma (2013) 30(23):1973–82 10.1089/neu.2013.3001 23822888 PMC3837503

[46] Lu XC Hartings JA Si Y Balbir A Cao Y Tortella FC Electrocortical pathology in a rat model of penetrating ballistic-like brain injury. J Neurotrauma (2011) 28(1):71–83 10.1089/neu.2010.1471 20964535

[47] Caboclo LO Neves RS Jardim AP Hamad AP Centeno RS Lancellotti CL Surgical and postmortem pathology studies: contribution for the investigation of temporal lobe epilepsy. Arq Neuropsiquiatr (2012) 70(12):945–52 10.1590/S0004-282X2012001200009 23295424

[48] Wieser HG ILAE Commission Report. Mesial temporal lobe epilepsy with hippocampal sclerosis. Epilepsia (2004) 45(6):695–714 10.1111/j.0013-9580.2004.09004.x 15144438

[49] Mathern GW Babb TL Vickrey BG Melendez M Pretorius JK Traumatic compared to non-traumatic clinical-pathologic associations in temporal lobe epilepsy. Epilepsy Res (1994) 19(2):129–39 10.1016/0920-1211(94)90023-X 7843168

[50] Pitkanen A McIntosh TK Animal models of post-traumatic epilepsy. J Neurotrauma (2006) 23(2):241–61 10.1089/neu.2006.23.241 16503807

[51] Kotapka MJ Graham DI Adams JH Gennarelli TA Hippocampal pathology in fatal non-missile human head injury. Acta Neuropathol (1992) 83(5):530–4 10.1007/BF00310031 1621508

[52] Maxwell WL Dhillon K Harper L Espin J MacIntosh TK Smith DH There is differential loss of pyramidal cells from the human hippocampus with survival after blunt head injury. J Neuropathol Exp Neurol (2003) 62(3):272–9 12638731 10.1093/jnen/62.3.272

[53] Blumcke I Beck H Lie AA Wiestler OD Molecular neuropathology of human mesial temporal lobe epilepsy. Epilepsy Res (1999) 36(2–3):205–23 10.1016/S0920-1211(99)00052-2 10515166

[54] Kovacs R Heinemann U Steinhauser C Mechanisms underlying blood-brain barrier dysfunction in brain pathology and epileptogenesis: role of astroglia. Epilepsia (2012) 53(Suppl 6):53–9 10.1111/j.1528-1167.2012.03703.x 23134496

[55] Cacheaux LP Ivens S David Y Lakhter AJ Bar-Klein G Shapira M Transcriptome profiling reveals TGF-beta signaling involvement in epileptogenesis. J Neurosci (2009) 29(28):8927–35 10.1523/JNEUROSCI.0430-09.2009 19605630 PMC2875073

[56] Ivens S Kaufer D Flores LP Bechmann I Zumsteg D Tomkins O TGF-beta receptor-mediated albumin uptake into astrocytes is involved in neocortical epileptogenesis. Brain (2007) 130(Pt 2):535–47 10.1093/brain/awl317 17121744

[57] Maxwell WL Pennington K MacKinnon MA Smith DH McIntosh TK Wilson JT Differential responses in three thalamic nuclei in moderately disabled, severely disabled and vegetative patients after blunt head injury. Brain (2004) 127(Pt 11):2470–8 10.1093/brain/awh294 15456707

[58] Salmond CH Chatfield DA Menon DK Pickard JD Sahakian BJ Cognitive sequelae of head injury: involvement of basal forebrain and associated structures. Brain (2005) 128(Pt 1):189–200 10.1093/brain/awh352 15548553

[59] Dixon CE Clifton GL Lighthall JW Yaghmai AA Hayes RL A controlled cortical impact model of traumatic brain injury in the rat. J Neurosci Methods (1991) 39(3):253–62 10.1016/0165-0270(91)90104-8 1787745

[60] Goodman JC Cherian L Bryan RM Jr Robertson CS Lateral cortical impact injury in rats: pathologic effects of varying cortical compression and impact velocity. J Neurotrauma (1994) 11(5):587–97 10.1089/neu.1994.11.587 7861450

[61] Hall ED Sullivan PG Gibson TR Pavel KM Thompson BM Scheff SW Spatial and temporal characteristics of neurodegeneration after controlled cortical impact in mice: more than a focal brain injury. J Neurotrauma (2005) 22(2):252–65 10.1089/neu.2005.22.252 15716631

[62] Anderson KJ Miller KM Fugaccia I Scheff SW Regional distribution of fluoro-jade B staining in the hippocampus following traumatic brain injury. Exp Neurol (2005) 193(1):125–30 10.1016/j.expneurol.2004.11.025 15817271

[63] Rola R Mizumatsu S Otsuka S Morhardt DR Noble-Haeusslein LJ Fishman K Alterations in hippocampal neurogenesis following traumatic brain injury in mice. Exp Neurol (2006) 202(1):189–99 10.1016/j.expneurol.2006.05.034 16876159

[64] Green RC Blume HW Kupferschmid SB Mesulam MM Alterations of hippocampal acetylcholinesterase in human temporal lobe epilepsy. Ann Neurol (1989) 26(3):347–51 10.1002/ana.410260307 2802534

[65] Garman RH Jenkins LW Switzer RC3rd Bauman RA Tong LC Swauger PV Blast exposure in rats with body shielding is characterized primarily by diffuse axonal injury. J Neurotrauma (2011) 28(6):947–59 10.1089/neu.2010.1540 21449683

[66] Svetlov SI Prima V Kirk DR Gutierrez H Curley KC Hayes RL Morphologic and biochemical characterization of brain injury in a model of controlled blast overpressure exposure. J Trauma (2010) 69(4):795–804 10.1097/TA.0b013e3181bbd885 20215974

[67] Bauman RA Ling G Tong L Januszkiewicz A Agoston D Delanerolle N An introductory characterization of a combat-casualty-care relevant swine model of closed head injury resulting from exposure to explosive blast. J Neurotrauma (2009) 26(6):841–60 10.1089/neu.2009-0898 19215189

[68] Lu J Ng KC Ling G Wu J Poon DJ Kan EM Effect of blast exposure on the brain structure and cognition in the *Macaca Fascicularis*. J Neurotrauma (2011):10.1089/neu.2010.1591 21639720

[69] Goldstein LE Fisher AM Tagge CA Zhang XL Velisek L Sullivan JA Chronic traumatic encephalopathy in blast-exposed military veterans and a blast neurotrauma mouse model. Sci Transl Med (2012) 4:134ra60 10.1126/scitranslmed.3003716 PMC3739428 22593173

[70] de Lanerolle NC Bandak F Kang D Li AY Du F Swauger P Characteristics of an explosive blast-induced brain injury in an experimental model. J Neuropathol Exp Neurol (2011) 70(11):1046–57 10.1097/NEN.0b013e318235bef2 22002430

[71] Clemedson CJ Criborn CO A detonation chamber for physiological blast research. J Aviat Med (1955) 26(5):373–81 13263274

[72] Säljö A Bao F Haglid KG Hansson HA Blast exposure causes redistribution of phosphorylated neurofilament subunits in neurons of the adult rat brain. J Neurotrauma (2000) 17(8):719–26 10.1089/089771500415454 10972247

[73] Risling M Plantman S Angeria M Rostami E Bellander BM Kirkegaard M Mechanisms of blast induced brain injuries, experimental studies in rats. Neuroimage (2011) 54(Suppl 1):S89–97 10.1016/j.neuroimage.2010.05.031 20493951

[74] Bass CR Panzer MB Rafaels KA Wood G Shridharani J Capehart B Brain Injuries from Blast. Ann Biomed Eng (2012) 40(1):185–202 10.1007/s10439-011-0424-0 22012085

[75] Reneer DV Hisel RD Hoffman JM Kryscio RJ Lusk BT Geddes JW A multi-mode shock tube for investigation of blast-induced traumatic brain injury. J Neurotrauma (2011) 28(1):95–104 10.1089/neu.2010.1513 21083431 PMC3019584

[76] Zhu F Mao H Dal Cengio Leonardi A Wagner C Chou C Jin X Development of an FE model of the rat head subjected to air shock loading. Stapp Car Crash J (2010) 54:211–25 21512910 10.4271/2010-22-0011

[77] Cernak I Merkle AC Koliatsos VE Bilik JM Luong QT Mahota TM The pathobiology of blast injuries and blast-induced neurotrauma as identified using a new experimental model of injury in mice. Neurobiol Dis (2011) 41(2):538–51 10.1016/j.nbd.2010.10.025 21074615

[78] Cernak I Savic J Malicevic Z Zunic G Radosevic P Ivanovic I Involvement of the central nervous system in the general response to pulmonary blast injury. J Trauma (1996) 40(3S):100S–4S 10.1097/00005373-199603001-00023 8606388

[79] Chavko M Prusaczyk WK McCarron RM Lung injury and recovery after exposure to blast overpressure. J Trauma (2006) 61(4):933–42 10.1097/01.ta.0000233742.75450.47 17033565

[80] Irwin RJ Lerner MR Bealer JF Mantor PC Brackett DJ Tuggle DW Shock after blast wave injury is caused by a vagally mediated reflex. J Trauma (1999) 47(1):105–10 10.1097/00005373-199907000-00023 10421195

[81] Säljö A Svensson B Mayorga M Hamberger A Bolouri H Low-level blasts raise intracranial pressure and impair cognitive function in rats. J Neurotrauma (2009) 26(8):1345–52 10.1089/neu.2008-0856 19317610

[82] Readnower RD Chavko M Adeeb S Conroy MD Pauly JR McCarron RM Increase in blood-brain barrier permeability, oxidative stress, and activated microglia in a rat model of blast-induced traumatic brain injury. J Neurosci Res (2010) 88(16):3530–9 10.1002/jnr.22510 20882564 PMC2965798

[83] Park E Gottlieb JJ Cheung B Shek PN Baker AJ A model of low-level primary blast brain trauma results in cytoskeletal proteolysis and chronic functional impairment in the absence of lung barotrauma. J Neurotrauma (2011) 28(3):343–57 10.1089/neu.2009.1050 21142686

[84] Axelsson H Hjelmqvist H Medin A Persson JK Suneson A Physiological changes in pigs exposed to a blast wave from a detonating high-explosive charge. Mil Med (2000) 165(2):119–26 10709373

[85] Cheng J Gu J Ma Y Yang T Kuang Y Li B Development of a rat model for studying blast-induced traumatic brain injury. J Neurol Sci (2010) 294(1–2):23–8 10.1016/j.jns.2010.04.010 20478573

[86] Nakagawa A Manley GT Gean AD Ohtani K Armonda R Tsukamoto A Mechanisms of primary blast-induced traumatic brain injury: insights from shock-wave research. J Neurotrauma (2011) 28(6):1101–19 10.1089/neu.2010.1442 21332411

[87] Uchihara T Silver diagnosis in neuropathology: principles, practice and revised interpretation. Acta Neuropathol (2007) 113(5):483–99 10.1007/s00401-007-0200-2 17401570 PMC1868652

[88] Gentleman SM Nash MJ Sweeting CJ Graham DI Roberts GW Beta-amyloid precursor protein (beta APP) as a marker for axonal injury after head injury. Neurosci Lett (1993) 160(2):139–44 10.1016/0304-3940(93)90398-5 8247344

[89] Sherriff FE Bridges LR Sivaloganathan S Early detection of axonal injury after human head trauma using immunocytochemistry for beta-amyloid precursor protein. Acta Neuropathol (1994) 87(1):55–62 10.1007/BF00386254 8140894

[90] Sherriff FE Bridges LR Gentleman SM Sivaloganathan S Wilson S Markers of axonal injury in post mortem human brain. Acta Neuropathol (1994) 88(5):433–9 10.1007/BF00389495 7847072

[91] Panickar KS Norenberg MD Astrocytes in cerebral ischemic injury: morphological and general considerations. Glia (2005) 50(4):287–98 10.1002/glia.20181 15846806

[92] Sofroniew MV Vinters HV Astrocytes: biology and pathology. Acta Neuropathol (2010) 119(1):7–35 10.1007/s00401-009-0619-8 20012068 PMC2799634

[93] Graeber MB Streit WJ Microglia: biology and pathology. Acta Neuropathol (2010) 119(1):89–105 10.1007/s00401-009-0622-0 20012873

[94] Gallyas F Wolff JR Böttcher H Záborszky L A reliable method for demonstrating axonal degeneration shortly after axotomy. Stain Technol (1980) 55(5):291–7 6162248 10.3109/10520298009067257

[95] Koliatsos VE Cernak I Xu L Song Y Savonenko A Crain BJ A mouse model of blast injury to brain: initial pathological, neuropathological, and behavioral characterization. J Neuropathol Exp Neurol (2011) 70(5):399–416 10.1097/NEN.0b013e3182189f06 21487304

[96] Krave U Al-Olama M Hansson HA Rotational acceleration closed head flexion trauma generates more extensive diffuse brain injury than extension trauma. J Neurotrauma (2011) 28(1):57–70 10.1089/neu.2010.1431 21047148

[97] Li XY Li J Feng DF Gu L Diffuse axonal injury induced by simultaneous moderate linear and angular head accelerations in rats. Neuroscience (2010) 169(1):357–69 10.1016/j.neuroscience.2010.04.075 20451584

[98] Omalu BI DeKosky ST Minster RL Kamboh MI Hamilton RL Wecht CH Chronic traumatic encephalopathy in a National Football League player. Neurosurgery (2005) 57(1):128–34 discussion 128-34, 10.1227/01.NEU.0000163407.92769.ED 15987548

[99] McKee AC Cantu RC Nowinski CJ Hedley-Whyte ET Gavett BE Budson AE Chronic traumatic encephalopathy in athletes: progressive tauopathy after repetitive head injury. J Neuropathol Exp Neurol (2009) 68(7):709–35 10.1097/NEN.0b013e3181a9d503 19535999 PMC2945234

[100] McKee AC Gavett BE Stern RA Nowinski CJ Cantu RC Kowall NW TDP-43 proteinopathy and motor neuron disease in chronic traumatic encephalopathy. J Neuropathol Exp Neurol (2010) 69(9):918–29 10.1097/NEN.0b013e3181ee7d85 20720505 PMC2951281

[101] Omalu B Hammers JL Bailes J Hamilton RL Kamboh MI Webster G Chronic traumatic encephalopathy in an Iraqi war veteran with posttraumatic stress disorder who committed suicide. Neurosurg Focus (2011) 31(5):E3 10.3171/2011.9.FOCUS11178 22044102

[102] Cullen DK Browne KD Xu Y Adeeb S Wolf JA McCarron RM Blast-induced color change in photonic crystals corresponds with brain pathology. J Neurotrauma (2011) 28(11):2307–18 10.1089/neu.2011.1718 22082449 PMC3218413

[103] Dalle Lucca JJ Chavko M Dubick MA Adeeb S Falabella MJ Slack JL Blast-induced moderate neurotrauma (BINT) elicits early complement activation and tumor necrosis factor alpha (TNFalpha) release in a rat brain. J Neurol Sci (2012) 318(1–2):146–54 10.1016/j.jns.2012.02.002 22537900

[104] Esiri M Perl D Oppenheimer’s Diagnostic Neuropathology. 3rd ed Boca Raton, FL: Taylor and Francis Group (2006). p. 15–83

[105] Abdel-Rahman A Abou-Donia S El-Masry E Shetty A Abou-Donia M Stress and combined exposure to low doses of pyridostigmine bromide, DEET, and permethrin produce neurochemical and neuropathological alterations in cerebral cortex, hippocampus, and cerebellum. J Toxicol Environ Health A (2004) 67(2):163–92 10.1080/15287390490264802 14675905

[106] Abdel-Rahman A Dechkovskaia AM Goldstein LB Bullman SH Khan W El-Masry EM Neurological deficits induced by malathion, DEET, and permethrin, alone or in combination in adult rats. J Toxicol Environ Health A (2004) 67(4):331–56 10.1080/15287390490273569 14713564

[107] Abdel-Rahman A Shetty AK Abou-Donia MB Disruption of the blood-brain barrier and neuronal cell death in cingulate cortex, dentate gyrus, thalamus, and hypothalamus in a rat model of Gulf-War syndrome. Neurobiol Dis (2002) 10(3):306–26 10.1006/nbdi.2002.0524 12270692

[108] Abdel-Rahman A Shetty AK Abou-Donia MB Subchronic dermal application of N,N-diethyl m-toluamide (DEET) and permethrin to adult rats, alone or in combination, causes diffuse neuronal cell death and cytoskeletal abnormalities in the cerebral cortex and the hippocampus, and Purkinje neuron loss in the cerebellum. Exp Neurol (2001) 172(1):153–71 10.1006/exnr.2001.7807 11681848

[109] Werner M Chott A Fabiano A Battifora H Effect of formalin tissue fixation and processing on immunohistochemistry. Am J Surg Pathol (2000) 24(7):1016–9 10.1097/00000478-200007000-00014 10895825

[110] Fix AS Garman RH Practical aspects of neuropathology: a technical guide for working with the nervous system. Toxicol Pathol (2000) 28(1):122–31 10.1177/019262330002800115 10668998

[111] Bolon B Anthony DC Butt M Dorman D Green MV Little PB “Current pathology techniques” symposium review: advances and issues in neuropathology. Toxicol Pathol (2008) 36(6):871–89 10.1177/0192623307312693

[112] Jortner BS The return of the dark neuron. A histological artifact complicating contemporary neurotoxicologic evaluation. Neurotoxicology (2006) 27(4):628–34 10.1016/j.neuro.2006.03.002 16650476

[113] Cammermeyer JI An evaluation of the significance of the “dark” neuron. Ergeb Anat Entwicklungsgesch (1962) 36:1–61 14018067

[114] Nelson JS Principles and Practice of Neuropathology. 2nd ed New York: Oxford University Press, Inc (2003). p. 1–21

[115] Graham DI Lantos PL Greenfield’s Neuropathology. 7th ed (Vol. 1). London: Arnold (2002). p. 123–74

[116] Gallyas F Güldner FH Zoltay G Wolff JR Golgi-like demonstration of “dark” neurons with an argyrophil III method for experimental neuropathology. Acta Neuropathol (1990) 79(6):620–8 10.1007/BF00294239 1694382

[117] Kherani ZS Auer RN Pharmacologic analysis of the mechanism of dark neuron production in cerebral cortex. Acta Neuropathol (2008) 116(4):447–52 10.1007/s00401-008-0386-y 18521615

[118] Kepes JJ Malone DG Griffin W Moral LA Yarde WL Jones S Surgical “touch artefacts” of the cerebral cortex. An experimental study with light and electron microscopic analysis. Clin Neuropathol (1995) 14(2):86–92 7606902

[119] Loberg EM Torvik A Distinction between artefactually shrunken and truly degenerated ‘dark’ neurons by in situ fixation with microwave irradiation. Neuropathol Appl Neurobiol (1993) 19(4):359–63 10.1111/j.1365-2990.1993.tb00452.x 8232757

[120] Csordas A Mazlo M Gallyas F Recovery versus death of “dark” (compacted) neurons in non-impaired parenchymal environment: light and electron microscopic observations. Acta Neuropathol (2003) 106(1):37–49 12665989 10.1007/s00401-003-0694-1

[121] Kovesdi E Pal J Gallyas F The fate of “dark” neurons produced by transient focal cerebral ischemia in a non-necrotic and non-excitotoxic environment: neurobiological aspects. Brain Res (2007) 1147:272–83 17349980 10.1016/j.brainres.2007.02.011

[122] Ishida K Shimizu H Hida H Urakawa S Ida K Nishino H Argyrophilic dark neurons represent various states of neuronal damage in brain insults: some come to die and others survive. Neuroscience (2004) 125(3):633–44 10.1016/j.neuroscience.2004.02.002 15099677

[123] Schmued LC Hopkins KJ Fluoro-Jade B A high affinity fluorescent marker for the localization of neuronal degeneration. Brain Res (2000) 874(2):123–30 10.1016/S0006-8993(00)02513-0 10960596

[124] Garman RH The return of the dark neuron. A histological artifact complicating contemporary neurotoxicologic evaluation. Neurotoxicology (2006) 27(6):1126 10.1016/j.neuro.2006.09.005 17049608

